# New Appliances and Things Medical

**Published:** 1906-04-07

**Authors:** 


### NEW APPLIANCES AND THINGS MEDICAL.

[We shall be glad to receive at our Office, 28 & 29 Southampton Street, Strand, London, W.C., from the manufacturers, specimens of all new preparations and appliances which may be brought out from time to time.]

### THE HOUSE OF LORDS WHISKY.—OLD HIGHLAND MALT WHISKY.

(James Saunders and Company, Limited, 15 Charlotte Street, Fitzroy Square, London, W., and Glasgow.)

This whisky is a thoroughly genuine all-malt pot-still whisky, perfectly matured, and we cannot conceive that any whisky could have better taste or effect than the one before us, or more worthy of all the attributes contained in its title. Such a whisky as Messrs. Saunders have supplied to the House of Lords for upwards of a quarter of a century can only be produced by prolonged maturation of the spirit, distilled from an all-malt mash in the pot-still. Immature pot-still whisky cannot be drunk as a pure whisky, nor can a blend derived solely from different pot-stills. Immature pot-still whisky can only be acceptable to the public when it is largely blended with grain spirit, and the function of the blender and of grain spirit is essentially to save maturation time. After the recent controversy pot-still distillers will soon learn that there are many other ways besides the blender and his grain spirit by which they can put on the market a cheap and wholesome all-malt pot-still whisky; but for the very best classes of whisky, such as Messrs. Saunders' House of Lords whisky, age, and age alone, will supply that which is required. We have no hesitation in strongly recommending this whisky, and adding our testimony to many others that it is a genuine, very old, all-malt, pot-still product.

### HUMANISED CONDENSED MILK.

(The Humanised Condensed Milk Company, Limited, 24 The Minories, London, E.C.)

We have before us a preparation, made in Norway, of milk which, when treated as directed on the tin, approximates closely in chemical composition to human milk, hence may correctly be termed humanised. The difficulty of at present obtaining humanised milk is such that there should be a good opening for a satisfactory preparation, supplied in a handy form. The milk as consumed by the child is prepared by adding one part of milk and two of water, and warming. It contains no preservative of any kind, and the tin is adequate to supply a day's diet to the infant. We are glad to see upon the tin a notice to the effect that the milk will not keep indefinitely when the tin is opened, and that should the finished product taste at all sour it should not be used. Statements of this kind help to educate an unreasonable public, and are distinctly to be encouraged. From what we have seen we regard the preparation as an excellent one.

### CREAM OF MALT WITH COD-LIVER OIL AND GUAIACOL CARBONATE.

(Oppenheimer, Son and Company, Limited, London.)

We have frequently in these columns drawn attention to the dietetic and therapeutic value of malt extracts, especially those as the one before us, which contain, in addition to malt, some active diastatic ferment. A preparation of this kind simply qua its malt extract moiety is a powerful aid to the digestion of amylaceous food, hence the extreme value of a nutritive tonic of this class in the mal-nutrition and dyspepsia of children and those suffering from tubercular affections. The substance before us, however, in addition to malt contains also cod-liver oil. The value of malt and cod-liver oil is so well known that it needs no emphasis, as also the peculiar fitness of cod-liver oil as a fat food in almost all wasting diseases. The addition of guaiacol carbonate to the above constituents greatly enlarges the substance's sphere of usefulness. This antiseptic, from its non-irritant properties, still holds the first place in the antiseptic treatment of pulmonary and intestinal tuberculosis, and its combination with a nutrient, thus saving the ingestion by the patient of a multiplicity of medicines, is highly to be recommended. The taste of the preparation is excellent, and we can say no more in praise of it than that it is fully up to the standard of the well-known firm which supply it.

